# Comparative fitness of reemerging St. Louis encephalitis virus in vertebrate and mosquito cells, *Culex tarsalis* and *Culex quinquefasciatus* mosquitoes, and mice

**DOI:** 10.1128/jvi.00952-26

**Published:** 2026-09-24

**Authors:** M. Arturo Flores Rodriguez, Hongwei Liu, Erik Turner, Elias Im, Rochelle Leung, Sunny An, Christopher M. Barker, Lark L. Coffey

**Affiliations:** 1 Department of Pathology, Microbiology, and Immunology, Weill School of Veterinary Medicine, University of California, Davis 240470, Davis, California, USA; 2 Freie Universität Berlin 9166, Berlin, Germany; The University of Texas Southwestern Medical Center, Dallas, Texas, USA

**Keywords:** vector-borne disease surveillance, collaborative cross mice, *Culex tarsalis* cells, duck embryonic fibroblast cells, Vero cells, viral growth kinetics, host-virus interactions, virus evolution, virus ecology, mosquito-borne virus

## Abstract

The human pathogenic orthoflavivirus St. Louis encephalitis virus (SLEV) reemerged in the western United States in 2015 after more than a decade of absence and has since expanded throughout California with sustained interannual transmission. This shift from the historically sporadic pattern of SLEV activity before 2003 raises the question of whether contemporary strains differ in fitness from earlier strains. To assess whether reemerging SLEV possesses enhanced infectivity or transmissibility, we compared five contemporary genotype III strains from California (2016–2023) with a historical genotype V strain from 2003. Growth kinetics were evaluated in mammalian, duck, and mosquito cells; vector competence was assessed in laboratory colonies of *Culex tarsalis* and *Culex quinquefasciatus* vectors; and SLEV RNA levels in blood were measured in collaborative cross recombinant intercross mice. Some genotype III strains produced higher titers than the historical genotype V strain in avian, but not mammalian or mosquito cells. Several genotype III strains infected and transmitted SLEV RNA more efficiently than the historical strain in both mosquito species, although no temporal trend in fitness was observed. SLEV fitness was comparable or greater in *Culex quinquefasciatus* than in *Culex tarsalis*. Sequencing identified no shared amino acid substitutions associated with vector infection phenotypes. Although genotype III strains exhibited a delayed peak relative to the historical strain, murine SLEV RNA levels in blood were comparable across strains. These findings show some contemporary strains exhibit equal or greater fitness than the historical strain, which may contribute to SLEV persistence and spread in California, underscoring the need for continued surveillance and targeted vector control.

**IMPORTANCE:**

St. Louis encephalitis virus (SLEV) reemerged in California in 2015 after more than a decade of absence and has since established sustained transmission and expanded geographically. The factors underlying this reemergence remain poorly understood. By comparing contemporary genotype III SLEV strains with a historical genotype V strain, we found that several contemporary strains exhibit equal or greater fitness compared to the historical strain in avian cells and are transmitted more efficiently by the two principal California vector species, *Culex tarsalis* and *Culex quinquefasciatus*. We also demonstrate that *Cx. quinquefasciatus* can transmit infectious genotype III SLEV, supporting its role in SLEV maintenance and spread. Despite differences in mosquito infection and transmission, we found no evidence that fitness in mosquito vectors or mice has continued to rise among strains detected more recently, suggesting that enhanced transmission is not driven by ongoing directional adaptation in these models. These findings indicate that contemporary genotype III SLEV strains possess transmission competence in mosquito vectors that may have contributed to successful reestablishment and persistence of SLEV in California. Improved understanding of the characteristics of reemerging SLEV strains can inform surveillance, risk assessment, and vector control efforts aimed at reducing human exposure to prevent disease caused by SLEV.

## INTRODUCTION

St. Louis encephalitis virus (SLEV, *Flaviviridae*, *Orthoflavivirus louisense*) circulates in an enzootic cycle between birds and mosquitoes in the Americas and can spill over into humans, causing febrile illness and sometimes encephalitis. SLEV produced periodic outbreaks in the United States (US) beginning in the 1930s and was a major cause of encephalitis during the 1970s (1, 2), including in California from the 1940s through the 1990s (3, 4). Because no specific treatment or vaccine for SLEV exists, mosquito control remains the primary public health tool for limiting SLEV transmission. In California, public health agencies monitor SLEV activity using human cases, sentinel chicken seroconversions, mosquito surveillance, and the distribution and abundance of mosquito vectors. Mosquito surveillance showed near-annual SLEV activity from 1970 until 2003 (3), the year the related flavivirus West Nile virus (WNV) invaded the state (5). Starting the next year (2004) until 2015, SLEV was not detected in California mosquitoes. The reasons for the disappearance of SLEV are not fully understood, but sterilizing cross-protection by WNV (6) and high levels of WNV herd immunity in shared avian reservoirs (7) may have quenched SLEV activity after WNV invaded. In 2015, a SLEV outbreak occurred in Phoenix, Arizona, concurrent with a WNV epidemic (8). That same year, SLEV was detected again in California and subsequently spread northward from southern California into the Central Valley and farther north from 2016 to 2025 (9) into 22 of 58 (38%) of California counties. Since 2015, SLEV-positive mosquito pools have included *Culex stigmatosoma*, *Culex pipiens*, *Culex tarsalis*, and *Culex quinquefasciatus* (10), the latter three of which were previously identified as the primary vectors of both WNV and historic SLEV in California (11–14). Between 2015 and 2025, 95% of SLEV-positive pools in California were *Cx. tarsalis* or *Cx. quinquefasciatus* (10). The geographic expansion, increasing number of human cases in most years, and detection of SLEV-positive mosquito pools since 2015 show SLEV is becoming increasingly prevalent in California (10). This pattern contrasts with historical pre-2003 SLEV activity, during which the virus was typically locally introduced, amplified for a 1–2 year period, and then disappeared (4, 15–17). Moreover, although SLEV and WNV now co-circulate in many of the same California counties, SLEV remains largely absent from WNV hotspots in the north and south and is instead more common in the Central and Coachella Valleys (9). The interannual persistence and geographic spread of reintroduced SLEV since 2015 raises the question of whether its reestablishment is mediated by increased fitness, defined here as infectivity in mammalian, duck, and mosquito cells, viremia in mice, and infectivity and transmissibility in two colonies of the primary California mosquito vector species, *Cx. tarsalis* and *Cx. quinquefasciatus*.

Our analyses (18, 19) comparing SLEV genomes in the western US from 2015 to 2018 with publicly available sequences show that these strains are most closely related to SLEV associated with an outbreak in Argentina in 2005 (20) and to a 2014 isolate from Argentine mosquitoes (21), supporting a South American introduction. SLEV circulating in California and the western US from 2014 to 2018 forms at least four geographically distinct clusters (a–d), all belonging to genotype III (19). Subsequent SLEV phylogenetic studies conducted after 2018 indicate that multiple SLEV importations have occurred in California, Arizona, and other western states; most genomes belong to genotype III (22); Texas also reports genotype II (23). To our knowledge, 2014 was the first time genotype III SLEV was detected in the US, although Bayesian phylogenetics suggest it was present in the US at least a year earlier, in 2013 (23). Despite these genetic insights, no phenotypic studies in cells, mosquito vectors, or mice have characterized infection or transmission dynamics of reemerging genotype III SLEV, nor compared post-2015 strains with pre-2004 SLEV (e.g., strains from 2003, which belong to genotype V that is not the direct ancestor of genotype III), that historically circulated in California and have been extensively evaluated in mosquito and avian hosts (7, 16, 24–29). We therefore sought to compare the relative fitness of reemerging post-2015 genotype III SLEV with pre-2003 genotype V, where genotype V was the last genotype detected in California prior to the 11-year absence of SLEV activity in mosquitoes. For context, WNV, which shares many avian reservoirs and *Cx*. vector species with SLEV, has evolved to increase transmission competence in *Cx*. spp. across the US since 2002 (30). Work in California further showed that WNV strains from later outbreaks (2004–2012) outcompete a 2003 strain in house finches but not in *Cx. tarsalis* (31, 32), suggesting that enhanced avian rather than mosquito fitness likely contributed to WNV amplification to outbreak levels during a decade of circulation in California. Although WNV evolution in both vectors and avian hosts across the US and in California is well documented, comparable studies for SLEV are lacking, leaving unanswered whether changes in viral fitness contributed to SLEV reemergence in California after 2015. To address this gap, the goal of this study was to evaluate the extent to which SLEV reemergence may be driven by altered replication and infectivity in mammalian, duck, and mosquito cells, as well as in collaborative cross recombinant intercross mice (RIX), and infectivity and transmissibility in two established colonies of primary California mosquito vectors. Mammalian African green monkey kidney cells were used to represent a standard arbovirus susceptible cell line and *Cx. tarsalis* cells were selected to represent a mosquito host. Although ducks are not recognized as amplifying hosts of SLEV, duck embryonic fibroblasts are susceptible to flaviviruses (25) and were selected as an avian cell line. The collaborative cross RIX mice were chosen as an immunocompetent mouse model of flavivirus infection and were used here to compare viral RNA levels in blood, a metric of viral fitness. Prior studies show RIX mice are susceptible to infection with WNV (33, 34) and tick-borne Powassan virus (35). Furthermore, in contrast to established murine SLEV models employing immunocompromised mice (36) and intracranial inoculation (37), we sought to use a mouse model that supports infection after peripheral inoculation. We compared the fitness of one historic SLEV strain from 2003 that is in genotype V and five SLEV genotype III strains from 2016 to 2023 in each of these systems.

## MATERIALS AND METHODS

### Biosafety

All work with SLEV was performed in a biosafety level 3 laboratory at the University of California, Davis, under approved Biological Use Authorization #R1863.

### Cell culture

African green monkey kidney (Vero, ATCC CCL-81) cells were used for SLEV isolation from mosquito pools, growth curves, and virus titrations from growth curves, bloodmeals, and mosquito saliva. Vero cells were maintained in Dulbecco’s Modified Eagle’s Medium (DMEM) supplemented with 5% heat-inactivated fetal bovine serum (FBS), 100 U/mL penicillin, and 100 μg/mL streptomycin at 37°C with 5% CO_2_ in a humidity-controlled incubator. Primary *Cx. tarsalis* (CxTr) were used for growth curve experiments. CxTr cells were a gift from Dr. Dane Jasperson at the United States Department of Agriculture. CxTr cells were maintained at 27°C in the dark in modified Schneider’s insect medium prepared from Schneider’s medium supplemented with sodium bicarbonate, L-glutamine, L-asparagine, reduced glutathione, and 10% heat-inactivated FBS. Media additions were filter-sterilized prior to use. CxTr cells were maintained as closed cultures without supplemental CO_2_ using vented-cap flasks. Duck embryonic fibroblast (DEF) cells (ATCC CCL-141) were used for growth curve experiments. DEF were maintained in DEF cell culture medium consisting of DMEM supplemented with 10% heat-inactivated FBS and 1% penicillin-streptomycin at 37°C with 5% CO_2_ in a humidity-controlled incubator.

### SLEV activity in California mosquitoes

The total annual number of SLEV-positive mosquito pools and SLEV detections in each mosquito species in California were sourced from the California Department of Public Health Vector Borne Disease Section Annual Reports from 2015 to 2024 (10). For the 2016 report, detailed data on the numbers of SLEV detections in different mosquito species were not provided; these data were instead derived from weekly bulletins from 2016 (38). The 2025 data were obtained from the WestNile.ca.gov website (39) and are current as of 8 December 2025.

### SLEV isolation from mosquito pools

This study focuses on contemporary SLEV from the period 2016 to 2023 and utilizes five genotype III SLEV strains isolated from California locations with ongoing mosquito pool activity compared to a historical genotype V strain from 2003. Pools of *Cx. tarsalis* and *Cx. quinquefasciatus* collected in California (Table 1) and confirmed SLEV-positive by an established qRT-PCR method (40) were obtained through routine arbovirus surveillance conducted by mosquito abatement districts in California and the Davis Arbovirus Research and Training Laboratory at the University of California, Davis. SLEV-positive mosquito pools were centrifuged at 4,000 × *g* for 5 min to pellet debris and then filtered through a 0.22 μM filter (Millex GP, Millipore, Billerica, MA, USA). The filtrate was inoculated onto confluent T25 Vero cell monolayers and incubated for 4 days. Virus recovered from culture supernatants was subsequently passaged 2–4 additional times on Vero cells to generate working stocks, which were titrated in Vero cells and stored at −70°C in aliquots until use. The genotype V SLEV strain isolated in 2003, representing the lineage historically circulating in California prior to 2004, was included as a comparator; this strain had been isolated previously and was re-sequenced since two additional Vero cell passages were performed to generate a working stock. The genotype III strains share ≈99% nucleotide and amino acid identity genome-wide with one another and ≈93% nucleotide and ≈98% amino acid identity with the 2003 genotype V strain (Table 2).

**TABLE 1:** SLEV strains used

Name	Strain identifier	Location	Year	Mosquito source	Passage history	GenBank accession number	Genotype
2003_V	IMP115	Imperial County, CA	2003	*Cx. tarsalis*	Vero P3	PX678850	V
2016_IIId	KERN345	Kern County, CA	2016	*Cx. quinquefasciatus*	Vero P4	MN233315	IIId
2017_IIIa	IMPR570	Imperial County, CA	2017	*Cx. tarsalis*	Vero P3	MN233313	IIIa
2022_IIId	FRWS339	Fresno West Side, CA	2022	*Cx. tarsalis*	Vero P2	PX678851	IIId
2023_IIIa	COAV3764	Coachella Valley, CA	2023	*Cx. tarsalis*	Vero P2	PX678852	IIIa
2023_IIId	MERC702	Merced, CA	2023	*Cx. tarsalis*	Vero P2	PX678853	IIId

**TABLE 2:** Genome-wide nucleotide/amino acid identity between SLEV strains used

Name	2003_V	2016_IIId	2017_IIIa	2022_IIId	2023_IIIa	2023_IIId
2003_V	100.0/100.0	93.04/98.69	93.03/98.75	93.15/98.72	93.07/98.72	93.16/98.69
2016_IIId	93.04/98.69	100.0/100.0	99.66/99.77	99.61/99.74	99.50/99.71	99.38/99.65
2017_IIIa	93.03/98.75	99.66/99.77	100.0/100.0	99.45/99.74	99.35/99.71	99.57/99.77
2022_IIId	93.15/98.72	99.61/99.74	99.45/99.74	100.0/100.0	99.79/99.80	99.20/99.68
2023_IIIa	93.07/98.72	99.50/99.71	99.35/99.71	99.79/99.80	100.0/100.0	99.10/99.65
2023_IIId	93.16/98.69	99.38/99.65	99.57/99.77	99.20/99.68	99.10/99.65	100.0/100.0

### SLEV growth curves

Vero, DEF, and CxTr cells were seeded in 24-well plates 1 day prior to SLEV inoculation to achieve approximately 80% confluence at the time of inoculation. Triplicate wells of cells were inoculated with SLEV strains at a multiplicity of infection (MOI) of 0.1 in 100 µL per well, which was incubated for 1 h at 37°C (Vero, DEF) or 27°C (CxTr) with rocking every 10 min. After the incubation, cells were washed twice with DPBS and overlaid with 1 mL of growth medium. Supernatants were collected at 0, 1, 2, 3, 4, 5, 6, and 7 days post-inoculation (dpi) by transferring 20 µL from each well into 180 µL of viral diluent consisting of DPBS with 1% FBS, followed by immediate storage at −70°C.

### SLEV titrations and infectious virus detection in saliva

Titers of SLEV stocks, growth curve samples, and bloodmeals were quantified by plaque assays using Vero cells. The presence of infectious virus was qualitatively assessed in a subset of randomly selected mosquito saliva samples that had detectable SLEV RNA. For SLEV stocks, growth curve samples, and bloodmeals, serial 10-fold dilutions of virus were inoculated onto confluent monolayers in 6-well plates. For saliva, confluent monolayers in 12-well plates were inoculated with 50 µL of mosquito saliva in DMEM. Inocula were adsorbed to cells for 1 h at 37°C with 5% CO_2_ with gentle rocking every 15 min. Following adsorption, an agarose-based overlay containing nutrient medium, 0.5% agarose, and 3% bicarbonate (Sigma-Aldrich, St. Louis, MO, USA) was added. After 5 days of incubation at 37°C and 5% CO_2_, a second overlay containing an additional 3% neutral red (Sigma Aldrich, St. Louis, MO, USA) was applied. On 7 dpi, plaques were visualized and, for stocks, growth curve samples, and bloodmeals, enumerated to determine the titer in log_10_ plaque-forming units (PFU)/mL. Saliva samples were tested once in one well due to volume constraints. Saliva samples are reported as positive if at least one plaque was detected. Each stock or bloodmeal titration was performed in duplicate, and the mean value is reported. SLEV titers reported from growth curves represent the mean titer from triplicate wells, where virus in each well was measured in one titration.

### Mosquitoes

Laboratory colonies of *Cx. tarsalis* and *Cx. quinquefasciatus* were used. The *Cx. tarsalis* colony originated from collections in 2002 at the Kern National Wildlife Refuge in Kern County, California. The *Cx. quinquefasciatus* colony (CQ1) originated from collections in Merced County, California, in the 1950s (41). For simplicity, we refer to the CQ1 colony as *Cx. quinquefasciatus,* but given the geographic origin of the colony within the zone of hybridization with *Cx. pipiens* (42), the CQ1 colony may represent an admixture of the two species. Both colonies have been maintained continuously since establishment. Mosquitoes were reared under insectary conditions of 22°C, ~30% relative humidity, and a 12 h:12 h light:dark cycle. Larvae were maintained in 1 L of deionized water at 200–400 larvae per pan and fed one pinch of fish food (Tetra, Melle, Germany) every other day until pupation. Adults were housed in 32.5 × 32.5 × 32.5 cm cages (BugDorm, #4F3030, Megaview Science, Taiwan) with constant access to 10% sucrose. Adults aged 3–7 days were used for vector competence experiments.

### Vector competence

A total of five genotype III SLEV strains were assessed; three were tested in *Cx. tarsalis*, and all were tested in *Cx. quinquefasciatus*. The historical 2003 genotype V strain was included for comparison in both species. Approximately 200 mixed-sex mosquitoes were aspirated from colony cages 1 day prior to bloodfeeding and transferred into 946 cm^3^-sized plastic containers with mesh lids and access to 10% sucrose. SLEV stocks were diluted in heparinized sheep blood (HemoStat Laboratories, Dixon, CA, USA) to target doses of 3, 5, and 6.7 log_10_ PFU/mL. The 3 and 5 log_10_ PFU/mL doses are within the range of avian reservoir viremias, whereas the 6.7 log_10_ PFU/mL dose exceeds avian SLEV viremias (43). Because infectious arbovirus titers can be affected by storage and freeze-thaw conditions (44), our use of previously frozen virus stocks may have reduced the effective infectious dose relative to the measured target titer, potentially bringing the highest dose closer to physiologically relevant avian viremia levels. Bloodmeals were offered for 60 min using a collagen membrane mounted on a Hemotek member feeder (Hemotek Ltd., Blackburn, United Kingdom) maintained at 37°C. Fully engorged females, identified by visible blood in the abdomen, were anesthetized for 10 s with CO_2_, sorted into clean plastic containers with mesh lids at a density of 30–60 mosquitoes, and held at 28°C with 60%–70% humidity and a 12 h:12 h light:dark cycle for 13, 14, or 15 days, with constant access to 10% sucrose. A total of 6 experiments were conducted in which 2–4 SLEV strains were compared at matched bloodmeal titers. Each strain was tested in 1–4 experiments; the combined data from all experiments are shown.

At 13, 14, or 15 days post-bloodfeeding, mosquitoes were CO_2_-anesthetized and placed immobile on a chill table for dissection. Harvests were distributed across the 3-day period due to personnel capacity constraints. Legs and wings were removed prior to saliva collection, which was performed by inserting the proboscis into a capillary tube containing FBS for 20–30 min. Each capillary tube was placed in a 1.5 mL tube containing 100 µL DMEM, and a 200 µL pipette tip was used to aspirate the FBS-containing saliva from the capillary tube. Bodies and legs and wings (the latter 2 tissues placed together) were placed into 2 mL tubes (Thermo Fisher Scientific, Emeryville, CA) containing 250 µL DMEM and a 5-mm glass bead (Thermo Fisher Scientific, Emeryville, CA). Dissection tools were rinsed once in 70% ethanol between each sample to prevent cross-contamination. Tissues were homogenized at 30 Hz for 4 min in a TissueLyser (Retsch, Haan, Germany) and centrifuged at 10,000 × *g* for 2 min, then stored at −70°C until processing.

### Mice

Collaborative cross RIX 024×023 mice were generated by the Systems Genetics Core Facility (SGCF) at the University of North Carolina. Triads of collaborative cross (CC) 024 and CC023 were used to generate F1 hybrids that were maintained at the highest health status barrier facility for breeding. The SGCF conducts yearly quality control genotyping, and individual ear punches from all mice used in this study were archived.

### Mouse infections

To compare replication kinetics of SLEV strains in blood, adult (8–12-week-old) collaborative cross RIX 024×023 mice were subcutaneously inoculated in the right rear footpad with 100 PFU of one of six SLEV strains or mock inoculum (DPBS), each diluted in Vero cell growth medium. The inoculum volume was 10 µL. Each experimental group consisted of 12 mice (6 males and 6 females). Mice were monitored daily for signs of clinical disease, including weight change relative to day 0 from 1 to 4 dpi, and neurologic signs, including ruffled fur, hunched posture, ataxia, and paralysis. Blood was collected via submandibular cheek bleed. To reduce repeated blood collection from individual animals, six mice per group were bled on 1 and 3 dpi, while a different set of six mice was bled on day 2 dpi. All mice were bled on day 4 dpi immediately prior to euthanasia. Four days post-inoculation was selected for euthanasia since the focus of these studies was the onset and early viremic period. Whole blood was immediately diluted 1:10 in DMEM and frozen at −70°C. Frozen diluted blood was thawed at room temperature prior to RNA extraction and RNAemia quantification by qRT-PCR.

### SLEV RNA detection, enumeration, and reporting

Tissues were thawed, and viral RNA was extracted from mouse blood and portions of bodies and legs and wings using the manufacturer’s instructions for the MagMax Viral RNA Extraction Kit (Thermo Fisher Scientific, Emeryville, CA, USA) or saliva using TRIzol LS (ThermoFisher). For TRIzol extractions, 400 µL TRIzol LS and 100 µL chloroform were added to 100 µL of saliva in DMEM. The tube was mixed vigorously and then centrifuged for 5 min at 20,000 × *g* at 4°C. The clear upper aqueous layer was transferred to a new 1.5 mL tube, and 200 µL of isopropanol was added. Then the tube was gently mixed by inverting 5–10 times, followed by incubation for 30 min at −20°C. The sample was centrifuged for 15 min at 20,000 × *g* at room temperature. The supernatant was discarded, and the sample was centrifuged for 30 s, and residual ethanol was removed using a 20 µL pipette. Total RNA isolated was suspended in 90 µL buffer following the MagMax manufacturer’s instructions (tissue samples) or 100 µL of nuclease-free water (saliva) from the TRIzol method. SLEV RNA in mosquito bodies, legs and wings, and saliva was quantified by qRT-PCR using a TaqMan Fast Virus 1-Step Mastermix and a SLEV-specific primer set (Table 3) at 20 μM using the following reagents: 3.2 μL TE buffer, 1.8 μL of the primer and probe mix, 5 μL of Fast mix, and 10 μL of RNA. The cycling conditions were as follows: 50°C for 5 min, 95°C for 20 s, and 40 cycles of 95°C for 3 s, followed by 60°C for 30 s. Cycle threshold (Ct) values were converted to RNA genome copies using standard curves generated from known SLEV RNA concentrations included on each qRT-PCR plate. At the consensus sequence level, all six SLEV strains are 100% identical across the forward primer and probe binding regions (Fig. S1). Both the forward primer and the probe exhibit a single nucleotide mismatch relative to all SLEV strains evaluated, with a second mismatch between the probe and 2003_V. Within the reverse primer-binding region, strains 2016_IIId and 2003_V each contain one nucleotide mismatch at distinct positions. The SLEV RNA of known concentration used to generate the standard curve was commercially procured from ATCC (Manassas, Virginia, Item# VR-3236SD), but the genomic sequence from which the primers and probe were designed is not reported by ATCC. Negative control samples lacking SLEV RNA were included in each run. Samples were assayed in technical duplicates or triplicates. Samples in which only one replicate produced a Ct <40 were considered negative. The limit of detection (LOD) was defined as the mean Ct for the least concentrated standard curve point producing a Ct <40 across all runs. Samples that did not yield a detectable Ct <40 are not included in group means for titer measurements. SLEV RNA concentrations are reported as log_10_ SLEV genomes/mL mouse blood or total mosquito tissue or saliva sample, calculated as geometric means and analyzed following log transformation. For vector competence studies, infection, dissemination, and transmission rates were calculated as the number of positive bodies, legs and wings, or saliva samples, respectively, divided by the number of total samples of the same type that were tested, multiplied by 100. Using a common denominator allows direct comparison across stages of vector competence and, for transmission, provides an epidemiologically relevant estimate of the probability that a mosquito will become capable of transmitting virus after ingesting an infectious blood meal.

**TABLE 3:** Primers used for SLEV RT-qPCR^*a*^

Primer name	Sequence 5′ to 3′
SLE2420 (forward)	CTGGCTGTCGGAGGGATTCT
SLE2487c (reverse)	TAGGTCAATTGCACATCCCG
SLE2444-probe (probe)	FAM-TCTGGCGACCAGCGTGCAAGCCG-BHQ

^*a*^ FAM, fluorescein amidite; BHQ, black hole quencher.

### SLEV genetic comparisons

Alignments of SLEV genomes and primers and probes were generated to compare nucleotide and amino acid identity using the EMBL-EBI MAFFT multiple sequence alignment tool and CLUSTAL (45).

### SLEV-positive mosquito pool mapping

Mosquito pool data were used with permission from reference 9 and mapped using ArcGIS Pro 3.1.

### Statistical analyses

All statistical analyses were performed in GraphPad Prism version 10.3.1. Analyses of infectious SLEV titers (PFU) and SLEV RNA levels were conducted on log-transformed values. Growth kinetics in cells and viremia kinetics in mice were compared among SLEV strains using a mixed-effects model, including strain, time, and their interaction, with cell replicate or mouse included as a random effect, and two-way ANOVA with Tukey’s multiple comparisons test. Area under the curve (AUC) was calculated on log_10_-transformed SLEV infectious virus measurements from growth curves. Ordinary one-way ANOVA was used to compare AUC. Two-sided Fisher exact pairwise comparisons with Holm correction were used to compare rates of infection, dissemination, and transmission between mosquito species and SLEV strains in cohorts of mosquitoes presented with the same bloodmeal titer. For RNA quantification, differences in mean RNA levels among SLEV strains in the same mosquito species were evaluated using ANOVA with Šídák’s multiple comparisons test. Only adjusted *P* values ≤ 0.05 were considered statistically significant. The specific statistical tests used for each comparison are provided in Results.

## RESULTS

**Fig 1:**
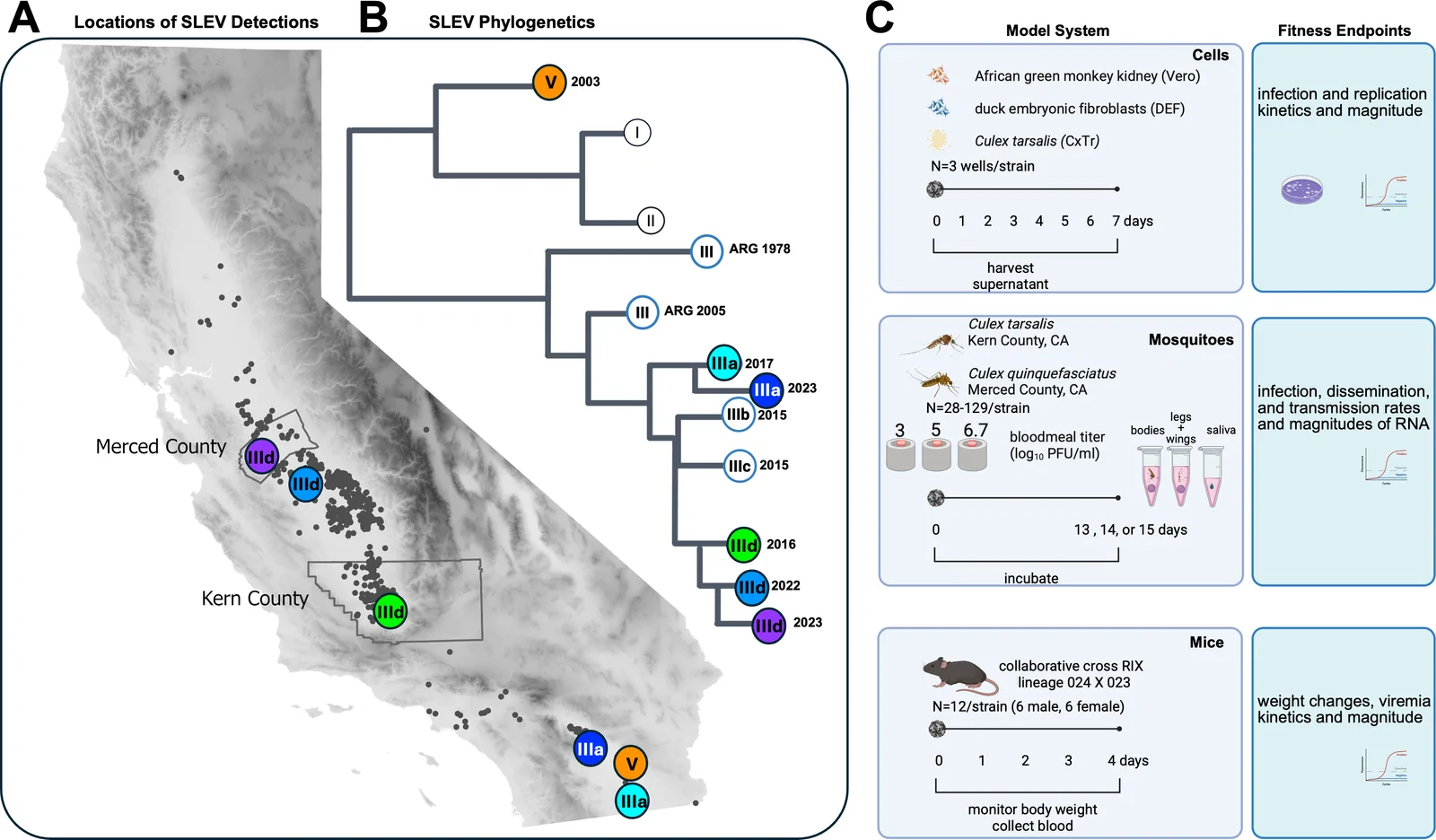
(**A**) Map of California showing locations of SLEV detections in mosquito pools from 2015 to 2025. The sources of the SLEV strains used in this study are shown in the colored circles and correspond to the labels on (**B**) the phylogenetic tree. ARG, Argentina. The map was created with ArcGIS Pro 3.1. (**C**) Experimental design for SLEV studies in three model systems: cells, colonized *Cx. tarsalis*, and *Cx. quinquefasciatus* mosquitoes from California, and collaborative cross RIX 024×023 mice. Fitness endpoints for each model are indicated. The image in panel C was created in BioRender (46).

SLEV was isolated from SLEV-positive mosquito pools collected across multiple regions of California (Fig. 1A). Contemporary isolates all belong to genotype III, contrasting with a historical California genotype V strain from 2003 (2003_V) (Fig. 1B). The 2003_V strain was included as a representative from the period preceding the 11-year gap in detected SLEV activity that began in 2004. We evaluated strain-specific fitness in three cell types (African green monkey kidney [Vero], DEF, and CxTr), two colonized *Cx*. vector species (*Cx. tarsalis* and *Cx. quinquefasciatus*), and one lineage (collaborative cross RIX 024×023) of immunocompetent adult mice (Fig. 1C). All virus strains were minimally passaged in Vero cells prior to use (Table 1).

### SLEV infection kinetics in cells

**Fig 2:**
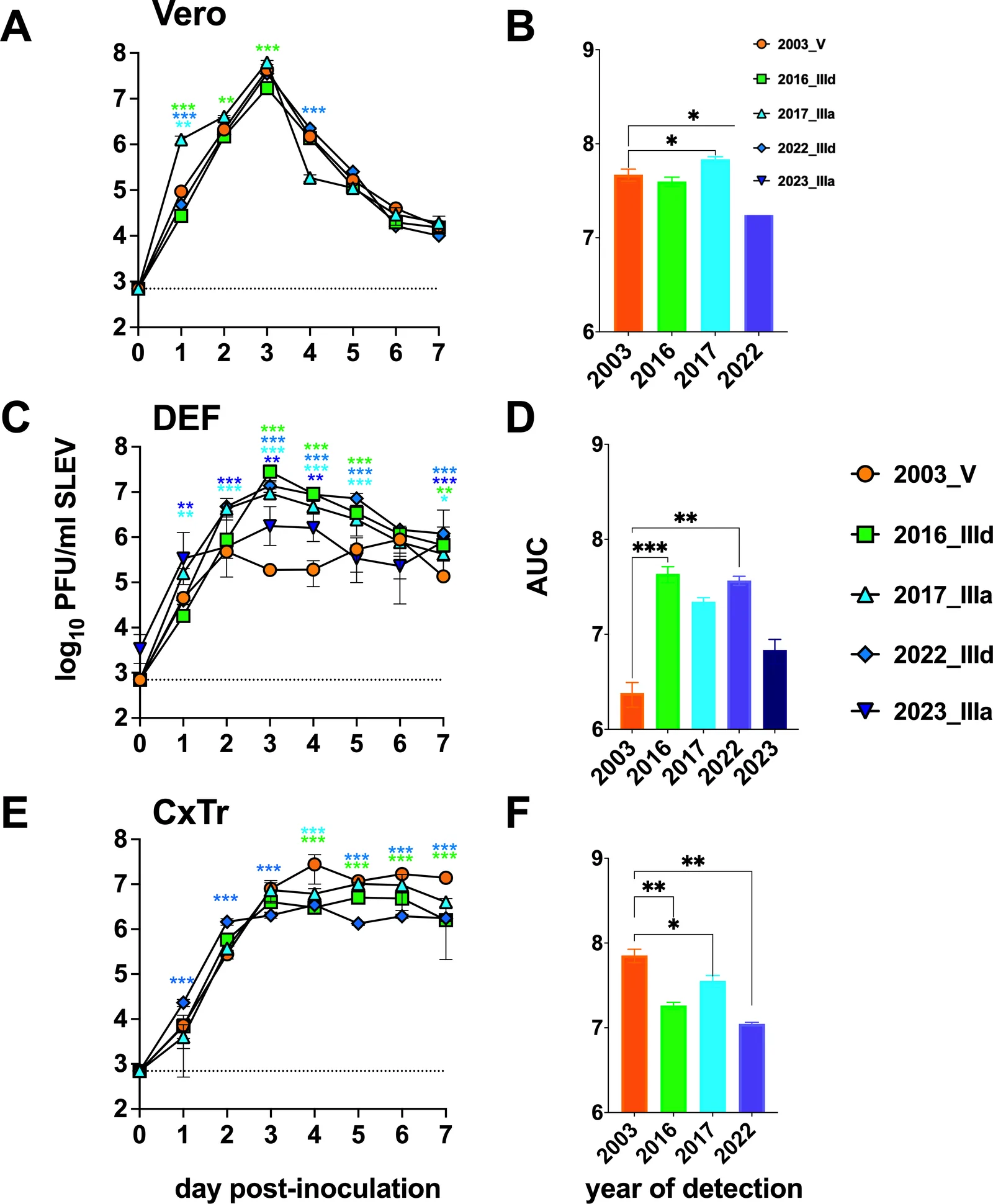
(**A, C, and E**) *In vitro* infection kinetics of SLEV strains in African green monkey kidney (Vero), Duck embryonic fibroblast (DEF), and *Cx. tarsalis* (CxTr) cells. An MOI of 0.1 was used. The log-transformed geometric mean virus titer from triplicate wells at each time point was plotted as PFU per milliliter. Error bars show geometric standard deviations. The dotted line represents the limit of detection, 2.8 log_10_ PFU/mL. Asterisks show comparisons of mean titers between 2003_V and other strains analyzed by mixed effects models with two-way ANOVA on log-transformed values. Colors of asterisks show which strain is different compared to 2003_V. (**B, D, and F**) AUC analysis showing mean AUC with error bars showing standard error of the mean. Asterisks show comparisons of mean AUC between 2003_V and other strains analyzed by ordinary one-way ANOVA on log-transformed values. * is *P* ≤ 0.01, ** is *P* ≤ 0.001, *** is *P* ≤ 0.0001.

We first assessed fitness of SLEV in cells by measuring infection kinetics over 7 days (Fig. 2). Fitness changes were detected as differences in titers or AUC for contemporary SLEV strains compared to 2003_V. In Vero cells (Fig. 2A), infection kinetics were similar among SLEV strains, with all viruses reaching mean peak titers of 7–8 log_10_ PFU/mL at 3 dpi. Mean titers for most genotype III strains were comparable to 2003_V, although strain 2017_IIIa produced significantly higher titers at earlier time points, from 1 to 3 dpi (mixed effects two-way ANOVA, *P* ≤ 0.0001), which was paralleled by significantly higher AUC (Fig. 2B). In DEF cells (Fig. 2C), most strains also reached peak titers at 3 dpi. All genotype III strains produced significantly higher mean DEF titers than 2003_V, particularly from 3 to 5 dpi (mixed effects two-way ANOVA, *P* ≤ 0.01), although no significant differences among strains were detected at 6 dpi. From 2 to 4 dpi, all genotype III strains reached mean titers at least 1 log_10_ PFU/mL higher than 2003_V in DEF cells. The AUC for strains 2016_IIId and 2022_IIId was significantly higher than 2003_V in DEF cells (Fig. 2D). In DEF cells, inter-strain variability in infection kinetics was greater than in Vero cells. In CxTr cells (Fig. 2E), peak titers were delayed relative to the two vertebrate cell lines, with maximum titers at 4 or 5 dpi. SLEV strains 2016_IIId and 2022_IIId produced significantly lower mean titers than 2003_V on multiple days (mixed effects two-way ANOVA, *P* ≤ 0.01), whereas 2017_IIIa produced lower titers compared to 2003_V at 4 dpi. The AUC of 2003_V was significantly higher than 2016_IIId, 2017_IIIa, and 2022_IIId in CxTr cells (Fig. 2F). Overall, these results indicate that some contemporary genotype III SLEV strains exhibit higher fitness than the historical 2003 genotype V strain in avian DEF, while infection differences are less pronounced in mammalian Vero and *Cx. tarsalis* mosquito cells.

### Dose response in mosquito vectors

**Fig 3:**
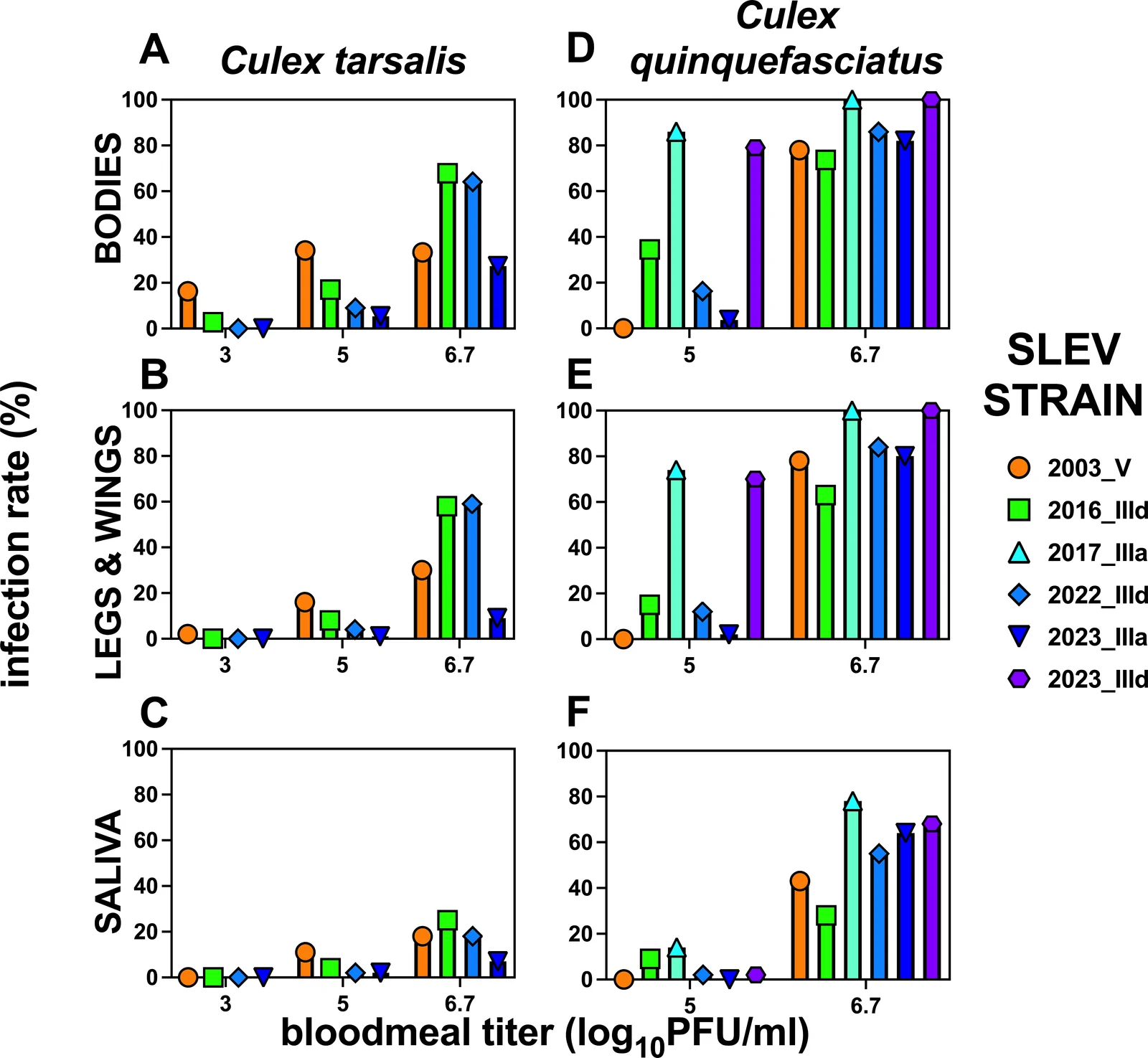
*Cx. tarsalis* (**A–C**) and *Cx. quinquefasciatus* (**D–F**) dose response to SLEV represented as infection (**A and D**), dissemination (**B and E**), and transmission (**C and F**) rates 13, 14, or 15 days post-bloodmeal in cohorts of mosquitoes that ingested bloodmeals containing different SLEV strains. Infection rates were calculated as the number of positive bodies, legs and wings, or saliva samples divided by the number of total samples of the same type that were tested, multiplied by 100. Each strain was tested in 1–4 experiments; the combined data from all experiments are shown. SLEV, St. Louis encephalitis virus; PFU, plaque-forming units.

We assessed the vector competence of the same five SLEV strains used in cell infections plus one additional 2023 strain (2023_IIId). We selected *Cx. tarsalis* and *Cx. quinquefasciatus* for evaluation because these species most frequently test positive for SLEV in California, representing 47% (1,289/2,731) and 48% (1,316/2,731), respectively, of SLEV mosquito pool detections statewide from 2015 to 2025 (Fig. S2). *Cx. tarsalis* were offered bloodmeals containing one of three SLEV doses, 3, 5, or 6.7 log_10_ PFU/mL, and *Cx. quinquefasciatus* were offered bloodmeals containing 5 or 6.7 log_10_ PFU/mL (Fig. 1C). Infection (bodies), dissemination (legs and wings), and transmission (saliva) rates, calculated as the fraction of positive samples out of the total number of samples of that tissue tested multiplied by 100, increased with bloodmeal titer for both species (Fig. 3; Table 4). At 3 log_10_ PFU/mL, infection rates were 0% or low for all strains in *Cx. tarsalis*. In contrast, infection, dissemination, and transmission rates increased when *Cx. tarsalis* ingested blood containing 5 or 6.7 log_10_ PFU/mL SLEV and in *Cx. quinquefasciatus* that ingested 6.7 versus 5 log_10_ PFU/mL. Back-titration confirmed that delivered bloodmeal doses were within ±0.5 log_10_ PFU/mL of the intended concentrations.

**TABLE 4:** Fractions and rates (%) of mosquito body, leg and wing, and saliva samples that tested positive for SLEV RNA in colonized California *Cx. tarsalis* and *Cx. quinquefasciatus* mosquitoes that ingested bloodmeals containing different SLEV strains

Species	BM titer (log_10_ PFU/mL)	Day(s)	SLEV strain	Infection		Dissemination		Transmission	
				Pos. body samples/total body samples tested (%)	Groups that differ statistically^*a*^	Pos. leg and wing samples/total leg and wing samples tested (%)	Groups that differ statistically^*a*^	Pos. saliva samples/total saliva samples tested (%)	Groups that differ statistically^*a*^
*Cx. tarsalis*	3	13, 14	2003_V	8/49 (16)	b	1/49 (2)	a	0/49 (0)	a
		13, 14	2016_IIId	1/36 (3)	ab	0/36 (0)	a	0/36 (0)	a
		13	2022_IIId	0/53 (0)	a	0/53 (0)	a	0/53 (0)	a
		13	2023_IIIa	0/48 (0)	a	0/48 (0)	a	0/48 (0)	a
	5	13, 14	2003_V	15/44 (34)	a	7/44 (16)	a	5/44 (11)	a
		13, 14	2016_IIId	9/53 (16)	ab	4/53 (8)	a	2/53 (4)	a
		14	2022_IIId	12/48 (25)	a	2/48 (4)	a	1/48 (2)	a
		14	2023_IIIa	3/56 (5)	b	1/56 (2)	a	1/56 (2)	a
	6.7	14	2003_V	11/33 (33)	ac	10/33 (30)	ab	6/33 (18)	a
		14	2016_IIId	19/28 (68)	b	16/28 (58)	a	7/28 (25)	a
		15	2022_IIId	25/39 (64)	ab	23/39 (59)	a	7/39 (18)	a
		15	2023_IIIa	12/44 (27)	c	9/44 (20)	b	3/44 (7)	a
*Cx. quinquefasciatus*	5	13	2003_V	0/66 (0)	b	0/66 (0)	c	0/66 (0)	a
		13	2016_IIId	19/55 (35)	a	8/55 (15)	a	5/55 (9)	ab
		13	2017_IIIa	55/64 (86)	c	37/50 (74)	b	7/50 (14)	b
		13	2022_IIId	7/43 (16)	ad	5/43 (12)	a	1/43 (2)	ab
		14	2023_IIIa	2/54 (4)	bd	1/54 (2)	ac	0/54 (0)	ab
		13	2023_IIId	85/108 (79)	c	35/50 (70)	b	1/50 (2)	ab
	6.7	14	2003_V	46/59 (78)	a	46/59 (78)	a	23/53 (43)	ac
		15	2016_IIId	42/57 (74)	a	36/57 (63)	a	16/57 (28)	c
		14	2017_IIIa	102/102 (100)	b	50/50 (100)	b	39/50 (78)	b
		15	2022_IIId	49/57 (86)	a	41/49 (84)	a	27/49 (55)	abc
		15	2023_IIIa	41/50 (82)	a	40/50 (80)	a	32/50 (64)	ab
		14	2023_IIId	129/129 (100)	b	50/50 (100)	b	34/50 (68)	ab

^*a*^ Letters show two-sided Fisher exact pairwise comparisons with Holm correction: values with no shared letter differ significantly at adjusted *P *< 0.05; values sharing at least one letter are not significantly different. BM, bloodmeal; Pos., positive; SLEV, St. Louis encephalitis virus; PFU, plaque-forming units; *Cx*., *Culex*.

### SLEV strain comparisons in mosquito vectors

We compared the relative fitness of SLEV strains in both mosquito species (Fig. 3; Table 4). In *Cx. tarsalis* at 3 log_10_ PFU/mL, all strains produced either no or low infection (Fig. 3A), and dissemination (Fig. 3B) and transmission (Fig. 3C) were similarly rare. At 5 log_10_ PFU/mL, infection rates of the 2003 genotype V strain were significantly higher than those of 2023_IIIa, while infection rates for 2003_V did not differ from those of 2016_IIId or 2022_IIId. The infection rate of 2022_IIId was also significantly higher than 2023_IIIa. Dissemination and transmission rates at this dose did not differ significantly among strains. At 6.7 log_10_ PFU/mL, infection rates of 2016_IIId were significantly higher than 2003_V and 2023_IIIa. No significant differences were observed in dissemination or transmission rates at this dose. Across doses, the two IIId lineage strains (2016_IIId and 2022_IIId) exhibited similar fitness profiles in all *Cx. tarsalis* tissues. Overall, these findings show strain-specific differences in SLEV fitness in *Cx. tarsalis*, with some but not all genotype III strains demonstrating higher infection rates than the historical genotype V strain, but without consistent differences in dissemination or transmission.

In *Cx. quinquefasciatus*, at 5 log_10_ PFU/mL, no mosquitoes became infected after ingesting the 2003 genotype V strain. In contrast, all five genotype III strains produced infection (Fig. 3D), ranging from 4% to 85%. At the 5 log_10_ PFU/mL dose, infection rates of 2017_IIIa were significantly higher than those of 2016_IIId and 2023_IIIa. Dissemination rates (Fig. 3E) for all strains except 2023_IIIa were higher than 2005_V. Strain 2017_IIIa disseminated significantly better than 2016_IIId, 2022_IIId, and 2023_IIIa. Only 2017_IIIa transmitted (Fig. 3F) significantly better than 2003_V. At 6.7 log_10_ PFU/mL, most *Cx. quinquefasciatus* mosquitoes exposed to any SLEV strain became infected, and numerous statistically significant differences were observed. The 2017_IIIa and 2023_IIId strains, which infected 100% of bodies, produced significantly higher infection rates than 2003_V. At the 6.7 log_10_ PFU/mL dose, dissemination rates for all strains were also high (63%–100%), and SLEV RNA in saliva was also frequently detected (28%–78%). Dissemination rates were significantly higher for 2017_IIIa and 2023_IIId compared to all other strains. Overall, genotype III SLEV strains demonstrated high and strain-specific fitness in both *Cx*. vector species, with several strains infecting and transmitting more efficiently than the historical 2003 genotype V strain. Despite this, there was no trend of increasing fitness associated with the year a strain was detected.

### SLEV RNA levels in mosquito tissues

**Fig 4:**
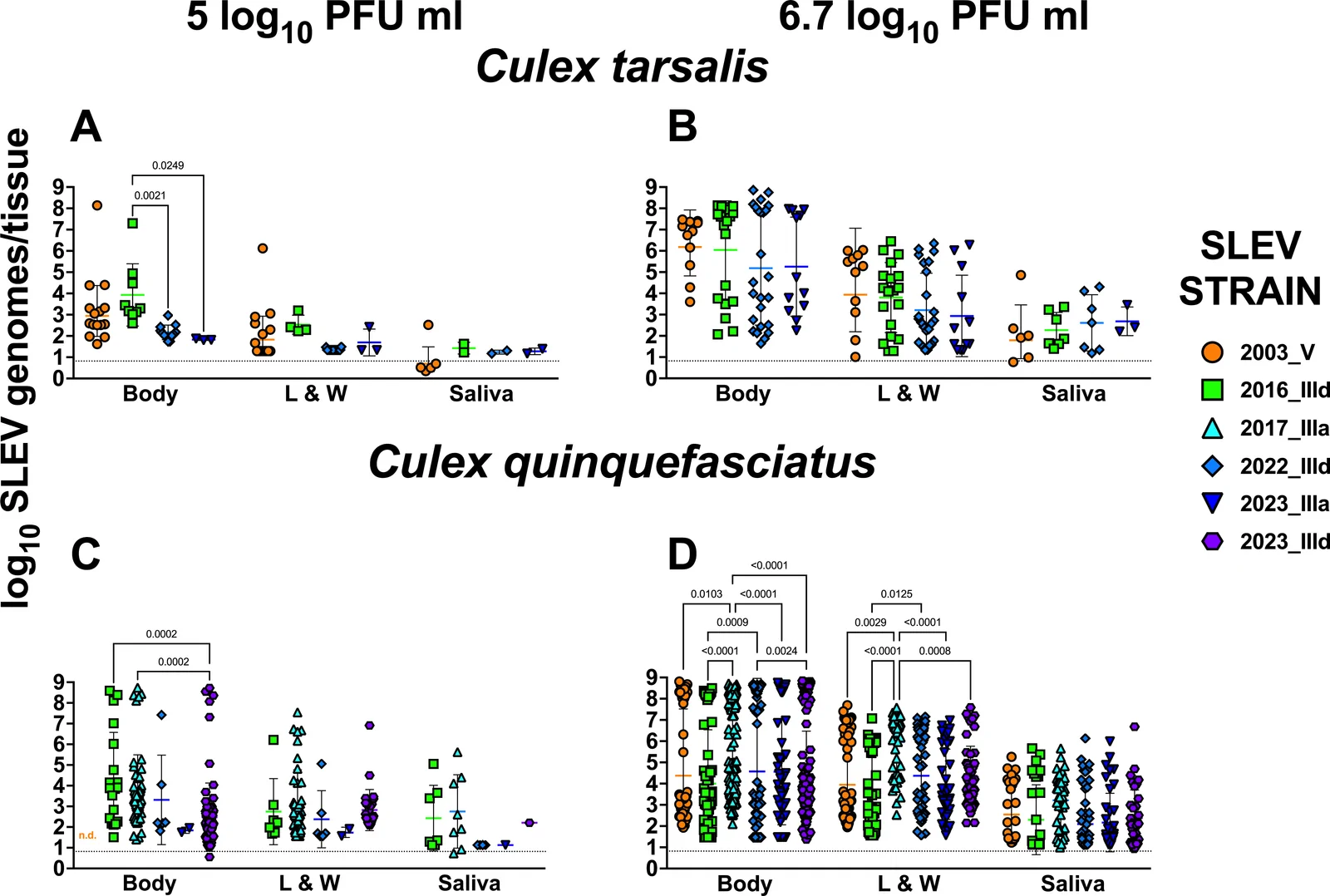
SLEV RNA levels 13, 14, or 15 days post-bloodmeal in individual *Cx. tarsalis* (**A and B**) and *Cx. quinquefasciatus* (**C and D**) mosquitoes that ingested 5 or 6.7 log_10_ PFU/mL of different SLEV strains. L & W represent legs and wings. Levels were not statistically significantly different unless indicated with a *P* value (two-way ANOVA, Šídák’s multiple comparisons test; only values with *P* ≤ 0.05 were considered significant). Solid horizontal lines show geometric means and whiskers show geometric standard deviations. The dotted line shows the mean LOD, 0.8 log_10_ genomes/tissue. The LOD for some samples with a detectable measurement was below the mean LOD, which explains why some samples are under the LOD line. n.d. denotes not detected.

We quantified SLEV RNA levels in individual infected mosquitoes from both species (Fig. 4). Mean titers decreased in the order bodies > legs and wings > saliva. In *Cx. tarsalis* at 5 log_10_ PFU/mL, mean RNA levels in bodies were significantly higher for 2016_IIId compared with 2022_IIId and 2023_IIIa (Fig. 4A). No significant differences in mean RNA levels were detected among strains in legs and wings or saliva at this dose. At 6.7 log_10_ PFU/mL, mean RNA levels in *Cx. tarsalis* did not differ significantly among strains in any tissue type (Fig. 4B). In *Cx. tarsalis*, mean RNA levels in any tissue for 2003_V were not significantly different compared to any other SLEV strain at both virus doses.

In *Cx. quinquefasciatus* at 5 log_10_ PFU/mL, mean RNA levels in bodies were significantly higher for 2016_IIId and 2017_IIIa compared with 2023_IIId (Fig. 4C). At 6.7 log_10_ PFU/mL, differences were observed across multiple strains in bodies and legs and wings (Fig. 4D), where mean RNA levels for 2017_IIIa were significantly higher than all other strains. No differences in mean RNA levels were detected in saliva from any strain at either dose. In *Cx. quinquefasciatus* at 6.7 log_10_ PFU/mL, mean RNA levels in bodies and legs and wings for 2003_V were significantly lower than 2017_IIIa but were not statistically different from any other SLEV strain. Together, these data show that SLEV RNA detection was tissue- and strain-dependent, with the highest viral RNA levels detected in bodies, limited strain-specific differences in *Cx. tarsalis*, more pronounced differences in *Cx. quinquefasciatus* (particularly at the highest dose), and no significant strain-associated variation in saliva RNA levels.

### SLEV infection detection in saliva

We next attempted detection of infectious SLEV in mosquito saliva (Table 5) as a surrogate for transmission to a vertebrate host, since measurement of SLEV RNA alone does not indicate infectivity. Undiluted saliva samples from a randomly selected subset of SLEV RNA-positive *Cx. quinquefasciatus* that ingested 6.7 log_10_ PFU/mL of either 2017_IIIa or 2023_IIId were tested using qualitative titrations. Plaque formation was observed in all saliva samples with SLEV RNA levels above 1.5 log_10_ genomes per sample. These results indicate that *Cx. quinquefasciatus* is capable of transmitting infectious genotype III SLEV.

**TABLE 5:** Infectious SLEV in *Cx. quinquefasciatus* saliva from mosquitoes that ingested 6.7 log_10_ PFU/mL in bloodmeals

SLEV strain	log_10_ SLEV genomes/saliva sample	Plaque formation
2017_IIIa	<0.8	−
	<0.8	−
	3.3	+
	3.8	+
	4.1	+
	4.2	+
	5.7	+
2023_IIId	1.5	−
	2.9	+
	3.9	+
	4.0	+
	4.2	+
	4.4	+
	4.7	+
	6.7	+

### Vector species comparisons

**Fig 5:**
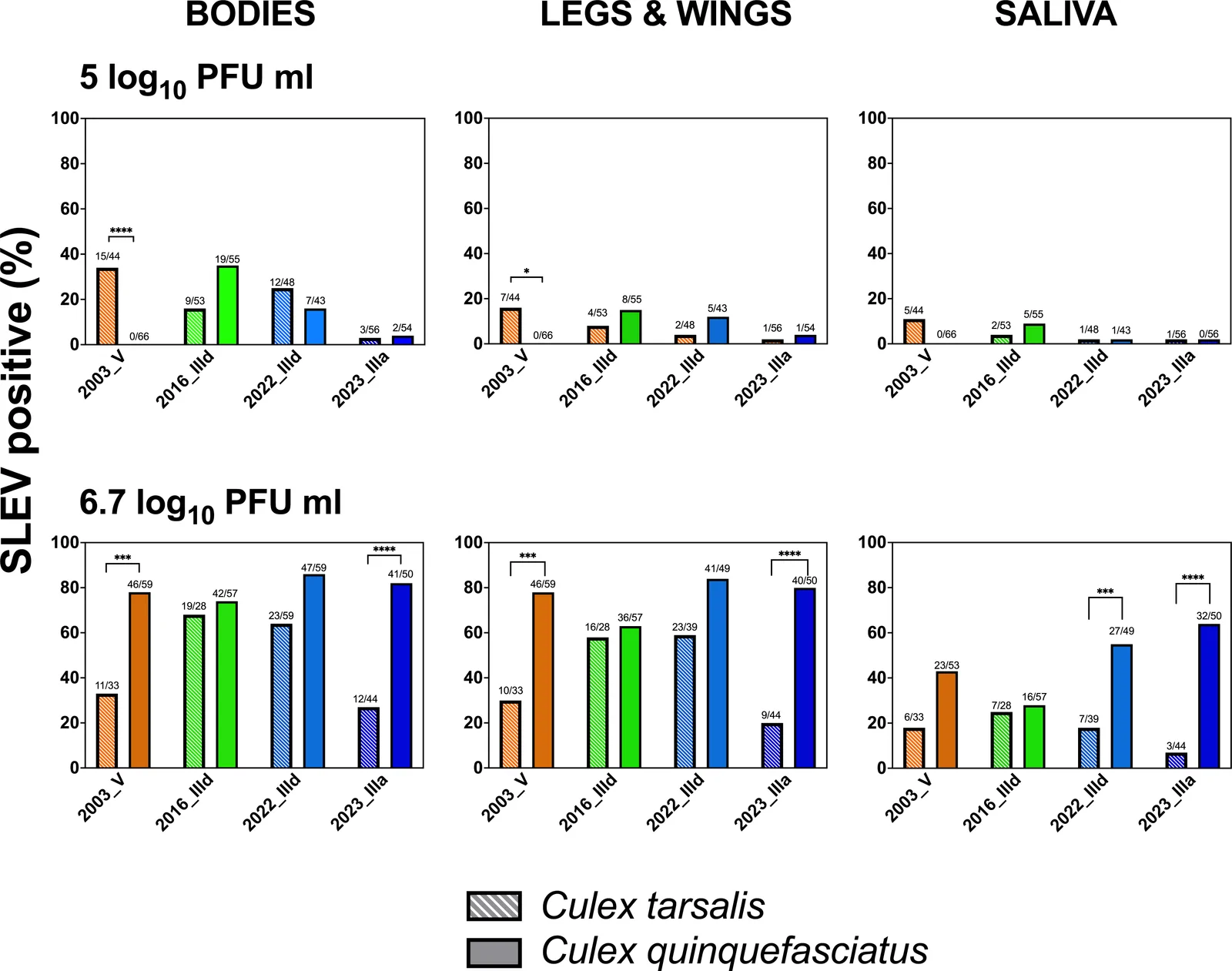
Rates of SLEV infection (bodies), dissemination (legs and wings), and transmission (saliva) by species 13, 14, or 15 days post-bloodmeal in cohorts of *Cx. tarsalis* and *Cx. quinquefasciatus* mosquitoes that ingested 5 or 6.7 log_10_ PFU/mL of different SLEV strains. Rates were calculated as the number of positive bodies, legs and wings, or saliva samples divided by the number of total samples of the same type that were tested, multiplied by 100. Rates were not statistically significantly different unless indicated with a symbol (Fisher’s exact tests; only values *P* ≤ 0.05 were considered significant, *** is *P* < 0.001, **** is *P* < 0.00001). SLEV, St. Louis encephalitis virus; PFU, plaque-forming units; *Cx*., *Culex*.

Using the same data from Fig. 3 and Table 4, we evaluated the relative fitness of SLEV across the two *Cx*. species by comparing infection, dissemination, and transmission rates at matched bloodmeal doses for the four strains that were evaluated in both species (Fig. 5). At 5 log_10_ PFU/mL, *Cx. quinquefasciatus* showed no susceptibility (0%, 0/66) to the 2003 genotype V strain, whereas *Cx. tarsalis* exhibited infection, dissemination, and transmission rates of 34% (15/44), 15% (7/44), and 11% (5/44), respectively. In contrast, for the three genotype III strains, infection, dissemination, and transmission rates at 5 log_10_ PFU/mL were not statistically different between the two species. At 6.7 log_10_ PFU/mL, infection rates in *Cx. quinquefasciatus* trended (2016_IIId, 2022_IIId) or were significantly (2003_V, 2023_IIIa) higher than in *Cx. tarsalis*. Dissemination and transmission rates also followed the same pattern where rates were higher in *Cx. quinquefasciatus* compared to *Cx. tarsalis*. Overall, these results indicate that the SLEV strains tested exhibit comparable or greater fitness in *Cx. quinquefasciatus* than *Cx. tarsalis*.

### SLEV genetic determinants of vector fitness

We compared genome-wide amino acid differences across strains (Table 6) and via alignment (Fig. S3) to evaluate whether variant amino acids are shared between strains with similar vector fitness (e.g., 2017_IIIa and 2023_IIId that infected the majority of *Cx. quinquefasciatus* at both 5 and 6.7 log_10_ PFU/mL doses). Of 60 amino acid differences across any of the six strains, 38 were unique to the 2003 V strain. There were no shared amino acid differences in strains with high (2017_IIIa and 2023_IIId) or low (2016_IIId, 2022_IIId, 2023_IIIa) *Cx. quinquefasciatus* infection patterns.

**TABLE 6:** Amino acid differences (*N* = 60) across six SLEV strains used for this study^*a*^

Gene	Genomic amino acid position	Gene amino acid position	2003_V	2016_IIId	2017_IIIa	2022_IIId	2023_IIIa	2023_IIId
C	10	10	R	K	K	K	K	K
	71	71	A	A	V	A	A	A
	92	92	L	M	L	L	L	L
	114	114	A	A	A	V	A	A
	116	116	I	I	I	T	I	I
prM	261	142	A	V	V	V	V	V
E	340	52	D	E	E	E	E	E
	352	64	T	S	S	S	S	S
	450	162	N	N	N	N	S	N
NS1	796	7	D	N	D	D	D	D
	859	70	N	S	S	S	S	S
	862	73	R	K	K	K	K	K
	885	96	H	Y	Y	Y	Y	Y
	887	98	R	K	K	K	K	K
	891	102	Q	R	R	R	R	R
	901	112	D	N	N	N	N	N
	963	174	G	E	E	E	E	E
	1077	288	T	T	T	T	I	T
NS2A	1236	33	L	F	F	F	F	F
	1250	47	I	V	I	I	I	I
	1291	88	C	Y	Y	Y	Y	Y
	1295	92	I	I	I	I	F	I
	1347	144	A	A	A	A	V	A
NS2B	1416	48	K	R	R	R	R	R
NS3	1837	338	A	A	A	T	A	A
	1853	354	S	N	N	N	N	N
	1898	399	K	K	K	K	R	K
	1978	479	N	N	S	N	N	N
	2049	550	V	V	V	A	V	A
	2088	589	R	K	K	K	K	K
	2093	594	R	K	K	K	K	K
NS4B	2184	67	T	T	T	T	T	A
	2195	78	G	S	S	S	S	S
	2210	93	L	S	S	S	S	S
NS5	2288	22	G	G	E	G	E	G
	2289	23	S	P	P	P	P	P
	2292	26	F	F	F	F	F	S
	2293	27	A	–	–	–	–	–
	2298	32	P	S	P	S	P	S
	2299_ins	33_ins	–	X	X	–	–	–
	2300	34	D	X	X	D	D	D
	2332	66	Q	Q	Q	Q	Q	H
	2343	77	A	S	S	S	S	S
	2395	129	V	I	I	I	I	I
	2418	152	A	V	A	A	A	A
	2513	247	V	I	I	I	I	I
	2649	383	Y	H	H	H	H	H
	2692	426	T	A	A	A	A	A
	2701	435	T	I	I	I	I	I
	2705	439	I	V	V	V	V	V
	2712	446	M	T	T	T	T	T
	2939	673	L	F	F	F	F	F
	2966	700	E	K	K	K	K	K
	2988	722	M	T	T	T	T	T
	3025	759	N	S	S	S	S	S
	3030	764	H	Y	Y	Y	Y	Y
	3119	853	V	I	I	I	I	I
	3161	895	V	I	I	I	I	I
	3199	933	I	T	T	T	T	T
	3278	1012	K	R	R	R	R	R
	3413	1147	I	V	V	V	V	A
	3429	1163	V	A	A	A	A	A

^*a*^ Grey shading shows the minority amino acid(s). “X” denotes incomplete sequence in GenBank record. ins, insertion; “–” denotes missing amino acid, C, capsid; prM, premembrane; M, membrane; E, envelope; NS, non-structural.

### SLEV kinetics in mice

**Fig 6:**
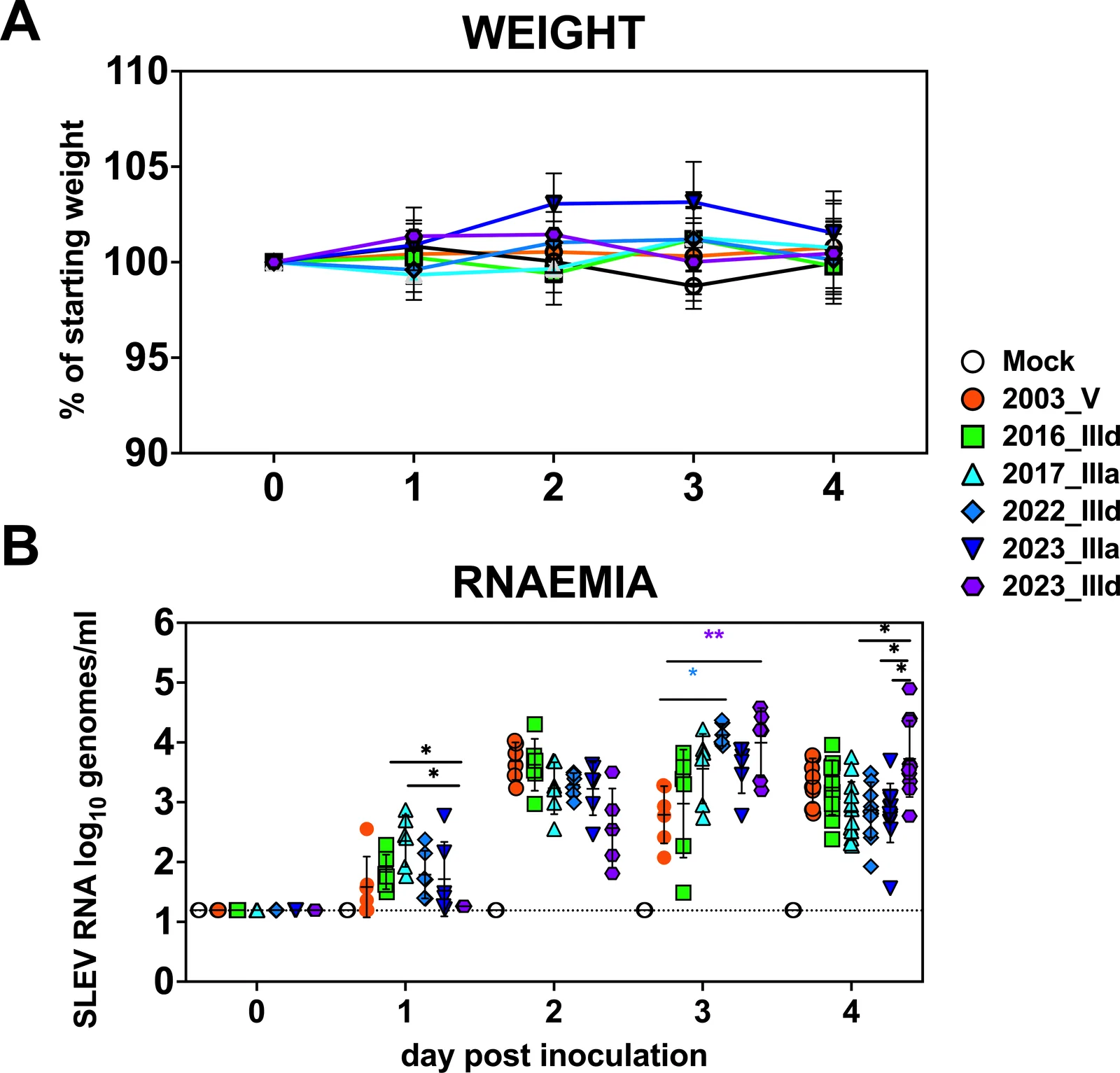
Comparison of SLEV strains in collaborative cross RIX 024×023 mice. Shown are (**A**) percent weight change and (**B**) viremia from 0 to 4 days post-inoculation. Each group included six male and six female mice. For panel B, viremia is presented as log-transformed geometric mean RNA levels in blood where each symbol shows an individual animal. To minimize blood collection from individuals, six mice were sampled on days 1 and 3, while a different set of six mice was sampled on day 2. All 12 mice were sampled on day 4, immediately before euthanasia. Error bars indicate geometric standard deviations. The dotted horizontal line denotes the limit of detection, 1.2 log_10_ SLEV genomes/mL blood. Statistical comparisons between strains were performed using mixed-effects models with two-way ANOVA on log-transformed values: * is *P* ≤ 0.01, ** is *P* ≤ 0.001. n.d., not detected. Data shown are from one experiment. Colored asterisks show which strain is different compared to 2003_V. Black asterisks show comparisons between genotype III strains.

Immunocompetent collaborative cross RIX mice serve as a model of flavivirus infection and were used here to compare SLEV RNA kinetics in blood across SLEV strains and to investigate the potential for clinical manifestations. The RIX 024×023 lineage was selected for this study because WNV-inoculated mice of this lineage consistently develop detectable viremia and some show clinical disease (33, 34). In our studies, none of the SLEV strains caused clinical disease in RIX 024×023 mice, and body weights remained within 5% of starting weight through the study endpoint at 4 dpi, similar to the mock-inoculated group (Fig. 6A). All six SLEV strains produced detectable SLEV RNA in blood (RNAemia) from 1 to 4 dpi, with peak RNA levels 2 or 3 dpi (Fig. 6B). The historical 2003_V strain peaked at 2 dpi, whereas most genotype III strains peaked at 3 dpi. Despite this difference in peak timing, mean SLEV RNA levels did not vary significantly between any strain and 2003_V at 1, 2, or 4 dpi. At 3 dpi, however, mean RNA levels for 2022_IIId and 2023_IIId were significantly higher than those for 2003_V. Some differences were also observed among genotype III strains: at 1 dpi, 2016_IIId and 2017_IIIa were significantly higher than 2023_IIId, whereas at 4 dpi, 2023_IIId had significantly higher RNA levels than 2017_IIIa, 2022_IIId, and 2023_IIIa. Overall, these data indicate that all SLEV strains produce subclinical infection in collaborative cross RIX 024×023 mice and similar RNA viremia kinetics, although most genotype III strains show a 1-day delay to peak viremia compared to the genotype V strain.

## DISCUSSION

SLEV reemergence in California after 2015 represents a striking departure from historical patterns, in which activity was sporadic, spatially limited, and often disappeared for years following introduction. The persistence and geographic expansion of genotype III SLEV across many California counties since its reemergence in 2015 raises questions about whether increased SLEV fitness may be contributing to this altered geographic pattern. By comparing contemporary California SLEV strains from 2016 to 2023 with a historical genotype V strain from 2003 that circulated prior to WNV invasion and the 11-year disappearance of SLEV statewide from 2004 to 2014, our study provides the first experimental evidence evaluating fitness of post-2015 SLEV in cells, mosquito vectors, and mice.

Several of the five post-2015 genotype III strains we evaluated showed equal or greater infectivity than the historical 2003 genotype V strain in duck embryonic fibroblast cells and in *Cx. quinquefasciatus*, particularly at the bloodmeal dose of 5 log_10_ PFU/mL. The 2003 strain and most genotype III strains achieved similar magnitudes of SLEV RNA in mosquito tissues. Notably, *Cx. quinquefasciatus* exposed to the 2003 strain at the 5 log_10_ PFU/mL dose did not become infected; by contrast, each genotype III strain produced measurable infection and, for some strains, dissemination and SLEV RNA in saliva, our proxy for transmission. This differential suggests that some contemporary SLEV lineages may be better suited to infect *Cx. quinquefasciatus* than 2003_V under ecologically relevant viremia levels typical of passerine avian reservoirs, which range from 2 to 6 log_10_ PFU/mL (43). Detection of infectious SLEV in saliva from *Cx. quinquefasciatus* for the two evaluated genotype III SLEV strains also shows that *Cx. quinquefasciatus* can transmit infectious genotype III SLEV. Given that *Cx. quinquefasciatus* is abundant in urban areas of southern and central California (47, 48), which continue to report much of the SLEV activity statewide in mosquitoes (10), enhanced vector competence in this species may facilitate sustained and geographically widespread transmission. In contrast, *Cx. tarsalis* displayed more heterogeneous and strain-specific responses to SLEV. Some genotype III strains (notably 2016_IIId and 2022_IIId) showed higher infection, dissemination, and transmission success than the 2003 strain at the 6.7 log_10_ PFU/mL dose, although no clear hierarchy of fitness emerged among strains or related to genetic classification within genotype III or detection year. This complexity mirrors earlier work on WNV in California, where viral fitness differences in *Cx. tarsalis* were sometimes inconsistent across strains and relatively small compared to those observed in avian hosts (31, 32). Taken together, our results suggest that while *Cx. tarsalis* remains a competent vector for both historical and contemporary SLEV, the enhanced fitness of genotype III strains is more evident, and potentially more epidemiologically consequential, in *Cx. quinquefasciatus*.

In contrast with the data from mosquito vectors, we observed no major differences in blood SLEV RNA magnitudes in collaborative cross RIX 024×023 mice. This suggests no fitness differences for the evaluated strains in a susceptible mammalian model of SLEV. Future studies comparing SLEV strain tissue tropism and differential immune responses in this mouse model, as well as fitness in avian reservoirs, will be important for determining whether strain-specific differences similar to our observations in duck cells contribute to modified SLEV transmission dynamics in natural avian-mosquito cycling.

Another important outcome is the absence of a temporal fitness trend among post-2015 SLEV strains. Despite concerns that ongoing circulation of SLEV since 2015 might select for increasingly infectious genotypes, our data do not support a directional shift in viral fitness. Instead, fitness differences among genotype III strains appear idiosyncratic and not associated with the year of detection. This pattern indicates that genetic variation within genotype III SLEV may influence fitness at a fine scale, but no single lineage among the strains we evaluated exhibits uniformly elevated infection or transmission potential in the evaluated model systems. Furthermore, the lack of shared mutations in SLEV strains with higher vector fitness suggests that varying vector competence is impacted by different unique mutations in individual strains or likely driven by factors other than viral genetic differences in the SLEV strains evaluated, including shared combinations of amino acid changes, synonymous mutations, non-coding or RNA folding patterns, or intrahost virus population dynamics.

This study has limitations. Cell culture studies can identify differences in flavivirus replication, but they rely on single or few (for CxTr) cell types that may not be physiologically relevant for arbovirus infection. Additionally, DEF cells are not representative of avian species known to amplify SLEV. Monocultures also have altered or antiviral responses and do not capture the barriers to infection, dissemination, transmission, and immune evasion encountered in vectors and vertebrate hosts. Consequently, *in vitro* replication phenotypes may not accurately predict epidemiologically relevant fitness or transmission potential. We also used long-established laboratory-adapted colonies of *Cx. tarsalis* and *Cx. quinquefasciatus* that may not reflect the genetic or ecological variation of wild California mosquitoes and may impact SLEV fitness. Recent changes in California vector populations may result in different findings, where distinct *Cx*. populations may vary in their fitness for SLEV. However, the feasibility of examining field-derived populations representative of the geographic and genetic diversity of these mosquito species in California proved outside the scope of this project. However, a prior study demonstrated that vector competence for WNV can vary over time within the same mosquito species (14), highlighting the value of using colonized mosquitoes derived from a single source population for direct comparisons of transmission phenotypes among virus strains. Furthermore, the use of a stable lab-adapted colony allows for a more ready comparison of future strains, while also acting as a common comparator for investigations into specific mosquito population-driven differences in vector competence. Local adaptation or hybridization within the *Cx. pipiens* complex could influence transmission outcomes for *Cx. quinquefasciatus* (which is part of the complex). Since we harvested mosquitoes starting on 12 days post-feeding, we may have missed kinetic changes at earlier time points, which could reveal earlier differences in fitness across strains. Transmission models show that a decreased extrinsic incubation period in mosquitoes changes the transmission dynamics of arboviruses, often producing a greater number of transmissions in a shorter period of time (49). Although collaborative cross RIX mice model flavivirus infection, they are not SLEV amplification hosts. Thus, viremia phenotypes should not be used to predict mosquito infectiousness or maintenance of avian-mosquito transmission cycles. Because mice were euthanized at 4 dpi, later viremia kinetic differences were not assessed. Although the SLEV strains evaluated represent the predominant genotype III lineages detected since 2015, limited sequencing of California SLEV since 2018 restricts our ability to assess whether these strains capture current viral diversity. Additional contemporary strains may display distinct vector fitness patterns. We also used a single genotype V comparator strain, which does not represent all genotype V SLEV. Use of additional strains may reveal variability between historic strains that is not apparent in our current analysis. Use of frozen rather than freshly cultured virus may have reduced infection (27), potentially underestimating infectivity, although comparisons among strains remain valid because all were tested under the same conditions. Mosquitoes exposed to different SLEV strains were harvested across a 1-day window (12–14 days post-exposure), but this is unlikely to have substantially affected infection or SLEV RNA levels based on relatively small kinetic changes in WNV over similar time periods (50–52). Saliva collected in capillary tubes provides only a surrogate measure of transmissibility because it does not replicate natural mosquito probing behavior or host factors influencing infection establishment. Vector competence experiments were conducted under controlled temperature and humidity conditions that do not reflect natural environmental variation, where temperature (51) and temperature variation (53) can alter infection dynamics. Except for a subset of saliva samples tested by infectious titrations, we quantified SLEV RNA rather than infectious virus in mosquitoes, potentially overestimating transmission potential if RNA detection does not correspond to infectivity (51). Finally, although dose-response experiments improve ecological relevance, artificial bloodmeal titers may not capture the full range of avian viremias; our (54) and others’ (55) work with other arboviruses and SLEV (56) show that artificial bloodmeals may misrepresent true infectiousness of vertebrate reservoirs for mosquitoes.

Our findings have implications for understanding SLEV ecology in California. The enhanced ability of some genotype III strains to infect *Cx. quinquefasciatus* may help explain why SLEV circulation has been concentrated in regions where this species is locally abundant, including the Coachella and Central Valleys of California. Besides increased mosquito vector competence, several other factors could contribute to SLEV reemergence. Expansion or movement of reservoir hosts and mosquito vectors into new areas, combined with environmental changes, such as warmer temperatures or altered water availability, that favor mosquito production and shorten the extrinsic incubation period, could increase transmission potential. Reemergence may also be driven by shifts in reservoir host demography, such as an influx of immunologically naïve birds, or by increased vertebrate competence in avian reservoirs. In addition, reduced cross-protection in WNV-immune birds could allow SLEV to circulate more effectively in bird populations. Finally, SLEV activity could reflect stochastic reintroduction and establishment events without major changes in viral traits or ecology, particularly if introduction coincides with favorable vector and host conditions.

This study provides phenotypic context for the genetic diversification of genotype III SLEV in the western US. As multiple genotype III lineages continue to circulate, defining how genetic variation translates into differences in virus fitness is important for forecasting areas of SLEV transmission and human disease risk. Future work based on more SLEV genomic data will integrate analyses of specific viral mutations that affect vector fitness and avian competence. Mixed-virus strain competition infections in mosquito vectors and avian reservoirs to minimize inter-host variability are also planned to further elucidate virological determinants underlying SLEV persistence in California in the post-WNV era.

## Data Availability

All data supporting the findings of this study are available as Data Set S1.

## SUPPLEMENTAL MATERIAL

Data Set S1 jvi.00952-26-s0001.xlsx Raw data.

Supplemental material jvi.00952-26-s0002.docx Figures S1 to S3.

ASM does not own the copyrights to Supplemental Material that may be linked to, or accessed through, an article. The authors have granted ASM a non-exclusive, world-wide license to publish the Supplemental Material files. Please contact the corresponding author directly for reuse.
