# Supplementary material for: Comparative fitness of reemerging St. Louis encephalitis virus in vertebrate and mosquito cells, Culex tarsalis and Culex quinquefasciatus mosquitoes, and mice
Source: J Virol. 2026 Sep 24;100(10):e00952-26. doi: 10.1128/jvi.00952-26 (PMC13712816; doi:10.1128/jvi.00952-26)
Supplement: Supplementary file 1 [file jvi.00952-26-s0002.docx]

**Supplemental Figure 1**: SLEV multiple sequence alignment showing primer (SLEV2420, SLEV2487c) and probe (SLE2444-probe) binding regions including single nucleotide polymorphisms. SLEV strain names denote year of isolation and genotype.

**Supplemental Figure 2**: SLEV positive mosquito pools in California from 2015-2025 by species. 2025 data is current as of December 8, 2025.

**Supplemental Figure 3**: Genome length amino acid alignment of six SLEV strains used in these studies. NS is non-structural. Dark shading shows locations of variant amino acids.
